# *Candida* antifungal drug resistance in sub-Saharan African populations: A systematic review

**DOI:** 10.12688/f1000research.10327.2

**Published:** 2017-01-20

**Authors:** Charlene Wilma Joyce Africa, Pedro Miguel dos Santos Abrantes

**Affiliations:** 1 Microbial Endogenous Infections (MEnIS) Research Laboratories, Department of Medical Biosciences, University of the Western Cape, Bellville, South Africa

**Keywords:** Candida, antifungal drug resistance, Africa

## Abstract

*Background*: *Candida* infections are responsible for increased morbidity and mortality rates in at-risk patients, especially in developing countries where there is limited access to antifungal drugs and a high burden of HIV co-infection. *Objectives: *This study aimed to identify antifungal drug resistance patterns within the subcontinent of Africa. *Methods*: A literature search was conducted on published studies that employed antifungal susceptibility testing on clinical *Candida* isolates from sub-Saharan African countries using Pubmed and Google Scholar. *Results*: A total of 21 studies from 8 countries constituted this review. Only studies conducted in sub-Saharan Africa and employing antifungal drug susceptibility testing were included. Regional differences in *Candida* species prevalence and resistance patterns were identified. *Discussion*: The outcomes of this review highlight the need for a revision of antifungal therapy guidelines in regions most affected by *Candida* drug resistance.  Better controls in antimicrobial drug distribution and the implementation of regional antimicrobial susceptibility surveillance programmes are required in order to reduce the high *Candida* drug resistance levels seen to be emerging in sub-Saharan Africa.

## Introduction

*Candida* species are known to shift from commensal to opportunistic infectious agents when triggered by factors such as immunosuppression, continuous usage of antibiotics and poor nutrition, leading to increased patient morbidity and mortality ^1– 3^. In severely immunocompromised patients, *Candida* species can spread through the bloodstream and gastrointestinal tract. This can lead to systemic candidiasis, with a reported mortality rate in developed countries of 38% ^4^ and 44% ^5^. *Candida* is currently the 4 ^th^ most commonly isolated microorganism in nosocomial bloodstream infections ^6^ and has been implicated in >78% of cancerous and precancerous oral lesions ^7^.

Various antifungal drugs with different modes of action have been developed over the years. These include polyene antifungals (e.g. nystatin and amphotericin B), which interfere with ergosterol synthesis, thereby causing cell membrane leakage; the imidazoles (e.g. miconazole, clotrimazole, econazole and ketoconazole), which also interfere with ergosterol and other cell membrane sterol synthesis; the echinocandins (e.g. anidulafungin, micafungin and caspofungin), which inhibit β 1–3 glucan synthesis, affecting the fungal cell wall and 5-flucytosine that in turn interferes with fungal RNA and DNA synthesis ^8^. The triazoles (including fluconazole, posaconazole, voriconazole and itraconazole) interfere with the synthesis of ergosterol and have been shown to have fewer side effects than some of the other antifungal drug classes ^9^.

Resistance to available antifungal therapies is widespread ^10, 11^, probably due to the widespread and repeated use of these drugs ^12^. Different *Candida* species have varying resistance patterns, which appear to be geographically determined ^13, 14^. Therefore early recognition of resistance facilitates the selection of an appropriate antifungal drug, with the use of oral antifungals in oropharyngeal candidiasis reserved for cases where there is no response to topical antifungal treatment ^15^. Resistance pattern surveillance to avoid an even higher number of improperly treated, and therefore resistant fungal infections, is imperative ^16^. This is a cause for concern in the case of immunocompromised patients, who are at a much higher risk of developing opportunistic complications. Importantly, sub-Saharan Africa is the region most affected by HIV, with approximately 25.8 million infected people in 2014 and accounting for almost 70% of the global number of new HIV infections ( http://www.who.int/mediacentre/factsheets/fs360/en/).

Programmes on species prevalence and antifungal surveillance have been successfully developed and introduced in Europe, Asia-Pacific, Latin America and North America ^17– 19^. The gap in antifungal drug resistance surveillance in Africa has been documented ^20^. Surveillance programmes are crucial tools in the transition from empirical antifungal treatment, which often does not work due to the diverse resistance levels seen in different regions, and the presence of species that are intrinsically resistant to certain antifungal drugs. The non-existence of routine diagnostics laboratories in most African countries has meant that many African patients are treated without the knowledge of which species they harbour and without any updated guideline data that could be used as a reference in prescribing antifungals. Possible causes for the lack of *Candida* surveillance programmes in Africa include lack of funding, the limited number of research collaborations and the existence of conflict areas within the continent. This prompted the need for a review of the current situation in Africa regarding the drug susceptibility profiles of *Candida* species available from different regions.

## Methods

### Data collection

A literature search was conducted on 21 published studies that employed antifungal susceptibility testing on clinical *Candida* isolates from 8 sub-Saharan African countries, with the aim of identifying antifungal drug resistance patterns within different regions of the subcontinent and included resistance data for 14 antifungal drugs. Searches were performed on PubMed and Google Scholar between August and November 2016 using the keywords ‘ *Candida*’, ‘Susceptibility Testing’, ‘Drug Resistance’ and ‘Antifungal’.

Data extracted from the individual studies included the regions within the different countries, patient health information, the methods used for antifungal susceptibility testing, the frequency of *Candida* species and their susceptibilities to antifungal drugs ( Dataset 1 ^21^).

### Inclusion criteria

We attempted to keep the review recent, and with the exception of a few earlier studies which provided an important comparison, only studies conducted in sub-Saharan Africa between the years 1998 and 2016 employing antifungal drug susceptibility testing were included.

### Exclusion criteria

Studies conducted in Africa which reported on the prevalence of *Candida* but did not describe antimicrobial susceptibility were excluded from this review.

## Results and discussion

The study populations included healthy ^22, 23^, HIV-positive ^22– 35^ and cancer patients ^23^, as well as patients with genitourinary tract infections ^36– 39^, respiratory tract infections ^32, 39^, meningitis ^39^ and candidemia ^40, 41^. Most studies relied on broth microdilution or disk diffusion for antimicrobial susceptibility testing, while one of the publications was a retrospective clinical study based on the patients’ response to antifungal therapy.

This review included seven studies from two regions in South Africa ^22– 25, 36, 40, 41^, three studies from different regions in Ethiopia ^33– 35^, three studies from two regions in Cameroon ^25– 27^, three studies from different regions in Nigeria ^28, 29, 37^, two studies from the same region in Ivory Coast ^30, 38^, one study from Tanzania ^31^, one study from Kenya ^32^ and one study from Ghana ^39^. Due to the paucity of studies and differences in isolation and antifungal susceptibility testing, a meta-analysis could not be conducted.

Non-albicans species, such as *C. glabrata* and *C. krusei* are reported to have innate resistance to antifungal drugs. *C. krusei* resistance has been reported from South Africa ^22, 25^, Cameroon ^25^, Nigeria ^28, 37^, Ghana ^39^, Tanzania ^31^ and Ethiopia ^34^. Initially thought to have innate resistance to azoles, *C*. *glabrata* resistance has been reported in Cameroon ^25^, Ethiopia ^34^ and Tanzania ^31^, while susceptibility has been reported in South Africa ^22, 25^ and Nigeria ^28^. This discrepancy may be explained by the phenotypic similarity between *C. glabrata, C. nivariensis* and *C. bracarensis,* which could possibly be confused in the absence of molecular typing methods and show different antifungal profiles ^42^. Resistant *C. glabrata* has increased in patients presenting with candidiasis in recent years ^43^, with increased mortality rates ^44^, and echinocandins have been recommended for the treatment of invasive *C. glabrata* infections showing resistance to azoles. However, co-resistance to both echinocandins and azoles in clinical isolates of *C. glabrata* have been reported ^45^, with two cases of echinocandin-resistant *C. glabrata* infections recently reported from South Africa ^36^.

A new multi-drug resistant species, *C. auris* is taking the world by storm. First discovered in Japan ^46^, this species has been found in nine other countries on four continents. The Centre for Disease Control and Prevention (CDC) has issued a warning for increased awareness of *C. auris* in healthcare settings. This nosocomial pathogen is frequently misdiagnosed, shows resistance to different classes of antifungals routinely administered, and is associated with high mortality rates ^47^. The isolation of this species in South Africa ^40^ appears to be the only report in Africa at the time of writing this paper.

Regional differences in *Candida* susceptibility profiles have been observed. ** In South Africa, earlier reports of baseline data demonstrated a high susceptibility (100%) of *C. albicans* to fluconazole, along with intrinsically resistant non-albicans species ^22, 24^, with more recent studies in South Africa showing an emerging resistance to azoles ^23, 25, 48^. The reasons for this change in susceptibility patterns is not clear, but it is worth noting that the earlier studies were done before the 2002 introduction of fluconazole as prophylaxis to patients attending HIV-AIDS clinics in South Africa ^49^.

Studies from abroad have reported cross-resistance to fluconazole in patients receiving itraconazole prophylaxis ^50^ and other previously administered azole therapies, such as ketoconazole and miconazole ^51, 52^. Similar cross-resistance was recently reported in South Africa where 37% of *C. parapsilosis* isolates were susceptible to fluconazole and voriconazole, and 44% of fluconazole-resistant isolates were voriconazole cross-resistant ^41^.

Studies from Bamenda ^25^ and Douala ^26^ in Cameroon showed high resistance of *C. albicans* isolates to azoles (>50% and 70% respectively), with low resistance reported from Mutegene ^27^ and Bamenda ^25^ for amphotericin B (4.9 and 4.3%) and 5-flucytosine (10.7 and 6.5%), respectively. The Douala study, on the other hand, reported increased *C. albicans* resistance to amphotericin B (52.6%) and 5-flucytosine (70%). A comparison of the Mutengene and Bamenda studies further revealed that *C. dubliniensis* and *C. tropicalis* susceptibilities differed between the two groups, with *C. dubliniensis* showing susceptibility to fluconazole and 100% resistance to amphotericin B in the Bamenda group, and increased resistance (66%) to fluconazole and no resistance to amphotericin B in the Mutengene group. *C. tropicalis* showed resistance to amphotericin B in the Bamenda group (50%) and only 4.3% resistance in the Mutegene group.

*C. albicans* resistance to amphotericin B has also been reported in Kenya (25.6%) ^32^ and Ghana (23.4%) ^39^, while no resistance was seen in studies from Ivory Coast ^30, 38^ nor Nigeria ^29^. Intermediate resistance values observed for clotrimazole and amphotericin B in studies from South West Cameroon, may indicate the need for administering higher doses to effectively treat these patients. This raises some concern, since both drugs are toxic at high concentrations and might have various side effects, such as the increase of blood pressure caused by clotrimazole therapy ^53^.

The application of topical antifungals, such as econazole and nystatin, is recommended for the localized treatment of *Candida* infections. *Candida* isolates from the Ivory Coast showed good susceptibility to nystatin with an increasing resistance noted in Ethiopia (1.3–4.7%), Kenya (36%), Gauteng, South Africa (67%) and Mutengene, Cameroon (68%). Resistance to econazole was reported in South West Cameroon ^26, 27^. Overall, *Candida* isolates from Eastern African countries demonstrated the lowest resistance levels, with the exception of Kenya where resistance values for clotrimazole (74%) and nystatin (35.6%) were high ^32^. Systemic antifungals are usually reserved for patients who are unresponsive to topical treatment in cases such as these.

Antifungal drug resistance of *Candida* species per region Click here for additional data file. Copyright: © 2017 Africa CWJ and Abrantes PMdS 2017 Data associated with the article are available under the terms of the Creative Commons Zero "No rights reserved" data waiver (CC0 1.0 Public domain dedication).

The prevalence of *Candida* species isolated from sub-Saharan Africa appeared to follow the same trend as in other regions, with *C. albicans* being the predominant species, followed by *C. glabrata*. However, in a study from Cameroon ^27^ and two studies from Nigeria ^28, 29^, *C. tropicalis* was the second most prevalent species. When comparing the drug resistance patterns with those in the rest of the world, *C. albicans* resistance to azoles was seen to be noticeably higher in Southwest South Africa ^25^ and two distinct regions in Cameroon ^25, 26^, while *C. glabrata* resistance to azoles was found to be generally low. However, in a study from South Africa ^41^, *C. glabrata* resistance to fluconazole was found to be similar to the high resistance levels seen in North America ^19^. *C. tropicalis* resistance to fluconazole was seen to be noticeably high in East African countries, with up to 50% resistance seen in Tanzania ^31^. Such high levels of resistance have not been documented in other regions.

Fluconazole is widely used in public health settings in the African continent and is used empirically in the treatment of systemic or localized *Candida* infections ^54^, as it is less toxic and regarded as more effective than imidazole antifungals, such as ketoconazole or amphotericin B, even though it is a teratogenic drug ^55, 56^. Although still somewhat effective in other regions, the use of azoles as first line drugs for systemic infection should be revisited in certain areas of South and West Africa, due to their increasing inefficacy. Regular monitoring of *Candida* at a regional level could therefore be an important tool to aid in the prescription of antifungals based on the prevalent species and their susceptibilities to antifungal drugs in areas where routine microbiological laboratory testing is not available.

The sale of antimicrobial medications is largely unregulated in Africa and is exacerbated by the influx of fake and adulterated drugs with little or no active ingredients, often available both in pharmacies and on the streets. This problem is aggravated by practitioners who prescribe antimicrobial medications empirically based on clinical presentation, without prior knowledge of which microbial agent(s) are causing infections in their patients. These issues pose a serious public health threat, as they are largely responsible for more and more antimicrobial drugs being rendered ineffective in treating life-threatening infections. This is especially true in the case of the antifungal armamentarium, which is already very limited ^57^, especially in resource-poor settings. Limitations of this study include the paucity of available data from African regions, differences in sample sourcing, as well as techniques for isolation and susceptibility profiles from different regions, all of which complicate a comparison of outcomes of the cited studies.

The regional differences in antifungal drug susceptibility of *Candida* species, often seen within the same country, are an important finding that justifies the implementation of *Candida* species prevalence and susceptibility testing programmes in the African continent, notably in at-risk population groups, such as HIV patients. With the emergence of inherently drug resistant non-albicans species, more studies on *Candida* prevalence and drug susceptibility are needed throughout sub-Saharan Africa. This is most critical in resource-poor areas where there is little or no information available, such as southern (with the exception of South Africa) and central African countries and countries bordering the Sahara.

We would like to conclude by adding that *Candida* identification to species level is rarely made in clinical settings in Africa, and patients are treated empirically based on their clinical symptoms. The introduction of routine antimicrobial susceptibility testing before initiation of therapy can be relatively expensive, but is certainly a long-term cost effective solution in preventing the progression of drug resistance. Changes in drug susceptibility over time serve as a reminder for the need to test clinical *Candida* isolates for sensitivity to antifungal drugs in the effort to improve patient care and reduce patient morbidity and mortality.

## Data availability

The data referenced by this article are under copyright with the following copyright statement: Copyright: © 2017 Africa CWJ and Abrantes PMdS

Data associated with the article are available under the terms of the Creative Commons Zero "No rights reserved" data waiver (CC0 1.0 Public domain dedication). 

**Dataset 1:** Antifungal drug resistance of *Candida* species per region. DOI, 10.5256/f1000research.10327.d145319 ^21^
