# Supplementary material for: Candida antifungal drug resistance in sub-Saharan African populations: A systematic review
Source: F1000Res. 2017 Jan 20;5:2832. Originally published 2016 Dec 8. [Version 2] doi: 10.12688/f1000research.10327.2 (PMC5247777; doi:10.12688/f1000research.10327.2)
Supplement: Antifungal drug resistance of Candida species per region [file f1000research-5-11516-s0000.tgz › 5d84bc74-cbe0-4003-9f42-cdc89f5ff46f_Dataset_1_Candida_AST_Review_paper.docx]

| **Author, Year** | **Region** | **Methodology** | **Species** | **Antifungal drug resistance (%)** | | | | | | | | | | | | | |
| --- | --- | --- | --- | --- | --- | --- | --- | --- | --- | --- | --- | --- | --- | --- | --- | --- | --- |
|  | | | | **Polyenes** | | **Fluorinated**  **pyrimidine analogue** | **Echinocandins** | | | **Triazoles** | | | | **Imidazoles** | | | |
| **South Africa** | | | | Amphotericin B | Nystatin | 5-Flucytosine | Anidulafungin | Caspofungin | Micafungin | Fluconazole | Itraconazole | Voriconazole | Posaconazole | Ketoconazole | Econazole | Miconazole | Clotrimazole |
| Blignaut *el al.*, 2000 | Gauteng, NE South Africa | Retrospective clinical study | N/A | _ | 66.6 | _ | _ | _ | _ | 0 | _ | _ | _ | _ | _ | 4.2 | _ |
| Blignaut *et al.*, 2002 | Gauteng, NE South Africa | Broth microdilution | *C. albicans* (n=466)  *C. dubliniensis* (n=10)  *C. glabrata* (n=9)  *C. krusei* (n=38)  *C. tropicalis* (n=9)  *C. parapsilopsis* (n=9)  *C. lusitaneae* (n=8)  *C. rugosa* (n=2)  *C. guillermondii* (n=2)  *C. kefyr* (n=1) | 8.4  0  55.6  23.7  44.4  0  0  0  0  0 | _ | 2.3  0  0  44.7  0  0  0  0  0  0 | _ | _ | _ | 0  0  0  100  0  0  0  0  0  0 | 0.4  0  11.1  21  0  0  0  0  0  0 | _ | _ | _ | _ | _ | _ |
| Abrantes *et al.*, 2014 | Cape Town, SW South Africa | Broth microdilution | *C. albicans* (n=106)  *C. dubliniensis* (n=10)  *C. glabrata* (n=12) | 8.5  10  41.7 | _ | 4.7  0  8.3 | 1.9  -  8.3 | 0  -  0 | 0  -  0 | 49.1  10  0 | 58.5  10  16.7 | 53.8  10  0 | _ | _ | _ | _ | _ |
| Magobo *et al*., 2014 | Various regions, South Africa | Broth microdilution | *C. auris* (n=4) | 0 | _ | _ | 0 | 0 | 0 | 100 | _ | _ | _ | _ | _ | _ | _ |
| Owotade *et al.*, 2016 | Johannesburg, NE South Africa | Broth microdilution | *C. albicans* (n=275) | 0 | _ | _ | 0.4 | 7.3 | 0 | 1.5 | 7.3 | 7.3 | 2.5 | _ | _ | _ | _ |
| Naicker *et al*., 2016 | Johannesburg, NE South Africa | Broth microdilution | *C. glabrata* (n=2) | _ | _ | _ | 100 | 100 (Only 1 isolate tested) | 100 | 0 (only 1 isolate tested) | _ | _ | _ | _ | _ | _ | _ |
| Govender *et al*., 2016 | Various regions, South Africa | Laboratory based surveillance study | *C. albicans* (n=521)  *C. glabrata* (n=108)  *C. krusei* (n=25)  *C. tropicalis* (n=58)  *C. parapsilopsis* (n=531) | _ | _ | _ | _ | _ | 0.6  1.9  0  1.7  0.2 | 0.2  8.3  -  3.4  53.1 | _ | 0.2  -  0  6.9  23.2 | _ | _ | _ | _ | _ |
| **Cameroon** | | | | | | | | | | | | | | | | | |
| Njunda *et al*., 2012 | Douala, SW Cameroon | Disk diffusion | *C. albicans* (n=175) | 52.6 | 13.1 | 70.9 | _ | _ | _ | 70.9 | _ | _ | _ | 12.6 | 13.7 | 30.3 | 38.3 |
| Njunda *et al*., 2013 | Mutengene, SW Cameroon | Broth microdilution | *C. albicans* (n=103)  *C. dubliniensis* (n=3)  *C. tropicalis* (n=23)  *C. famata* (n=4) | 4.9  0  4.3  0 | 73.8  100  56.5  50 | 10.7  66.7  8.7  25 | _ | _ | _ | 15.5  66.7  8.7  0 | 9.7  0  0  0 | 3.9  0  4.3  0 | _ | 1.9  0  0  0 | 7.8  0  21.7  0 | 8.7  0  13  0 | 10.7  33.3  21.7  0 |
| Abrantes *et al*., 2014 | Bamenda, NW Cameroon | Broth microdilution | *C. albicans* (n=92)  *C. dubliniensis* (n=1)  *C. glabrata* (n=24)  *C. krusei* (n=3)  *C. tropicalis* (n=4)  *C. para/lusi* (n=2) | 4.3  100  4.2  66.7  50  50 | _ | 6.5  0  0  0  0  0 | 0  -  12.5  0  0  - | 0  -  4.2  0  0  - | 0  -  66.7  0  0  - | 50  0  4.2  66.7  0  0 | 51  0  16.7  0  0  0 | 50  0  4.2  33.3  0  0 | _ | _ | _ | _ | _ |
| **Nigeria** | | | | | | | | | | | | | | | | | |
| Enwuru *et al*., 2008 | Lagos, SW Nigeria | Disk diffusion | *C. albicans* (n=30)  *C. dubliniensis* (n=1)  *C. glabrata* (n=4)  *C. krusei* (n=5)  *C. tropicalis* (n=13)  *C. parapsilopsis* (n=3)  *C. famata* (n=3)  *C. guillermondii* (n=1)  *C. kefyr* (n=5) | _ | _ | _ | _ | _ | _ | 10  0  0  40  7.7  0  0  0  0 | _ | _ | _ | _ | _ | _ | _ |
| Akortha *et al*., 2009 | Benin city, Southern Nigeria | Broth microdilution | *C. albicans* (n=138)  *C. glabrata* (n=68)  *C. krusei* (n=6)  *C. tropicalis* (n=4) | _ | _ | _ | _ | _ | _ | 4.3  0  100  0 | _ | _ | _ | 7.2  2.9  50  0 | _ | _ | _ |
| Nweze and Ogbonnaya, 2011 | SE Nigeria | Broth microdilution | *C. albicans* (n=54)  *C. dubliniensis* (n=9)  *C. tropicalis* (n=22)  *C.parapsilopsis*(n=18)  *C.guillermondii*(n=11) | 0  0  0  0  0 | _ | 9.5  11.1  9.1  11.1  0 | _ | _ | _ | 16.7  33.3  0  11.1  0 | 11.1  11.1  0  8.3  33.3 | 1.9  0  7.7  0  0 | _ | _ | _ | _ | _ |
| **Ivory Coast** | | | | | | | | | | | | | | | | | |
| Nébavi *et al*., 1998 | Abidjan, SE Ivory Coast | N/A | *C. albicans* (n=67) | 0 | 0 | _ | _ | _ | _ | _ | _ | _ | _ | 0 | _ | _ | _ |
| Djohan *et al*., 2012 | Abidjan, SE Ivory Coast | Semi solid medium microdilution | *C. albicans* (n=45) | 0 | _ | 0 | _ | _ | _ | 2.2 | 22.2 | 11.1 | _ | _ | _ | _ | _ |
| **Ghana** | | | | | | | | | | | | | | | | | |
| Feglo and Narkwa, 2012 | Kumasi, SW Ghana | Semi solid medium microdilution | *C. albicans* (n=33)  *C. dubliniensis* (n=4)  *C. glabrata* (n=12)  *C. krusei* (n=3)  *C. tropicalis* (n=8)  *C. parapsilopsis* (n=1)  *C. sake* (n=2)  *C. guillermondii* (n=1) | 27.3  0  0.8  66.7  25  0  0  0 | _ | 0.9  0  0  33.3  0  0  0  0 | _ | _ | _ | 0  0  0  100  0  0  0  0 | 0.6  0  25  0  0  0  0  0 | 0  0  0  0  0  0  0  0 | _ | _ | _ | _ | _ |
| **Tanzania** | | | | | | | | | | | | | | | | | |
| Hamza *et al*., 2008 | Dar Es Salaam, Eastern Tanzania | Broth microdilution | *C. albicans* (n=250)  *C. dubliniensis* (n=1)  *C. glabrata* (n=20)  *C. krusei* (n=10)  *C. tropicalis* (n=8)  *C. kefyr* (n=3)  *C. pintolopesii* (n=1) | _ | _ | _ | _ | _ | _ | 0  0  5  100  50  0  0 | 4  0  55  0  37.5  0  1 | _ | _ | _ | _ | _ | _ |
| **Kenya** | | | | | | | | | | | | | | | | | |
| Bii *et al*., 2002 | Nairobi, SW Kenya | Broth dilution | *C. albicans* (n=90) | 25.6 | 35.6 | 7.8 | _ | _ | _ | _ | _ | _ | _ | _ | _ | _ | 74.4 |
| **Ethiopia** | | | | | | | | | | | | | | | | | |
| Wabe *et al*., 2011 | Jimma, SE Ethiopia | Broth microdilution | *C. albicans* (n=42) | 2.3 | 4.7 | _ | _ | _ | _ | 11.9 | _ | _ | _ | 7.1 | _ | 7.1 | _ |
| Mulu *et al*., 2013 | Gondar, NW Ethiopia | Broth dilution | *C. albicans* (n=25)  *C. glabrata* (n=25)  *C. krusei* (n=10)  *C. tropicalis* (n=25)  Other spp. (n=5) | 4  -  -  -  - | _ | 4  4  -  4  - | _ | _ | 4  -  -  -  - | 16  4  10  8  20 | 8  4  -  4  - | _ | _ | -  4  10  -  20 | _ | _ | _ |
| Moges *et al*., 2016 | Addis Ababa, Central Ethiopia | Disk diffusion | *C. albicans* (n=106)  *C. glabrata* (n=24)  *C. krusei* (n=4)  *C. tropicalis* (n=17)  *C. kefyr* (n=2) | 1.9  8.3  25  17.6  50 | 0  4.2  0  0  50 | _ | _ | _ | _ | 6.6  16.6  50  35.3  0 | _ | _ | _ | 5.7  12.5  0  17.6  0 | _ | 0.9  0  0  0  0 | 0.9  4.2  25  5.9  0 |

“-“: drug not tested / breakpoint not available
