# Supplementary material for: Candida antifungal drug resistance in sub-Saharan African populations: A systematic review
Source: F1000Res. 2017 Jan 20;5:2832. Originally published 2016 Dec 8. [Version 2] doi: 10.12688/f1000research.10327.2 (PMC5247777; doi:10.12688/f1000research.10327.2)
Supplement: Peer review history

# Peer review: Referee response for version 1

**DOI:** 10.5256/f1000research.11123.r19187

**Published:** 2017-01-11

**Authors:** Roland N. Ndip

**Affiliations:** 1 Department of Microbiology and Parasitology, Faculty of Science, University of Buea, Buea, Cameroon

The title is appropriate for the content of the work; and the abstract captures a very good summary of the respective sections of the work. To me it is well written.

  The content is excellently presented with a very good and balanced literature review. All the articles cited are not only current, but very relevant to the scope of the work. The gaps in knowledge in this area were well highlighted and a good attempt made to bridge the gap.

 The conclusions are in line with the results obtained, and have profound recommendations which will improve on antifungal antimicrobial chemotherapy in sub-Saharan Africa.

 However, I have some minor questions, which I think would improve on the presentation if addressed.

 In the methods section under inclusion criteria: I was wondering why only studies conducted between 1998 and 2016 were used? Authors should state reasons to justify the stated period.

 Except for journal instructions, I am of the opinion that merging Results and Discussion as a single section to read: Results/Discussion will be better since so much discussion has been taken under the results section.

I have read this submission. I believe that I have an appropriate level of expertise to confirm that it is of an acceptable scientific standard.

# Peer review: Referee response for version 1

**DOI:** 10.5256/f1000research.11123.r18772

**Published:** 2017-01-06

**Authors:** António Paulo Gouveia de Almeida

**Affiliations:** 1 The Institute for Tropical Medicine, Universidade NOVA de Lisboa, Lisbon, Portugal; 2 Faculty of Health Sciences, University of Pretoria, Pretoria, South Africa

This is a very good and much needed review. The subject in question, *Candida* antifungal drug resistance in sub-Saharan Africa, is most pertinent and up to date.

 I would, however, suggest that, similarly to what authors do in the Introduction, they would mention in Discussion, how is the situation of *Candida* antifungal drug resistance in sub-Saharan Africa different from that in the rest of the world, in a general outlook and as a result from this literature review.

I have read this submission. I believe that I have an appropriate level of expertise to confirm that it is of an acceptable scientific standard.
